# Insights Into Mitochondrial Dynamics in Chlamydial Infection

**DOI:** 10.3389/fcimb.2022.835181

**Published:** 2022-03-07

**Authors:** Yewei Yang, Wenbo Lei, Lanhua Zhao, Yating Wen, Zhongyu Li

**Affiliations:** Hunan Provincial Key Laboratory for Special Pathogens Prevention and Control, Hunan Province Cooperative Innovation Center for Molecular Target New Drug Study, Institute of Pathogenic Biology, Hengyang Medical School, University of South China, Hengyang, China

**Keywords:** *Chlamydia*, mitochondrial dynamics, DRP1, P53, ATP

## Abstract

Mitochondria are intracellular organelles that are instrumental in the creation of energy, metabolism, apoptosis, and intrinsic immunity. Mitochondria exhibit an extraordinarily high degree of flexibility, and are constantly undergoing dynamic fusion and fission changes. *Chlamydia* is an intracellular bacterium that causes serious health problems in both humans and animals. Due to a deficiency of multiple metabolic enzymes, these pathogenic bacteria are highly dependent on their eukaryotic host cells, resulting in a close link between *Chlamydia* infection and host cell mitochondria. Indeed, *Chlamydia* increase mitochondrial fusion by inhibiting the activation of dynein-related protein 1 (DRP1), which can regulate host cell metabolism for extra energy. Additionally, *Chlamydia* can inhibit mitochondrial fission by blocking DRP1 oligomerization, preventing host cell apoptosis. These mechanisms are critical for maintaining a favorable environment for reproduction and growth of *Chlamydia*. This review discusses the molecular mechanisms of mitochondrial fusion and fission, as well as the mechanisms by which *Chlamydia* infection alters the mitochondrial dynamics and the prospects of limiting chlamydial development by altering mitochondrial dynamics.

## Introduction

For a long time, mitochondria were merely considered as cell energy factories, generating large quantities of ATP through oxidative phosphorylation (McBride et al., 2006). Subsequent research unraveled a pivotal role of mitochondria in several biological processes, including apoptosis, innate immunity, autophagy, redox signaling, calcium homeostasis, and stem cell reprogramming (Wakabayashi, 1999; Zhou et al., 2008; De Stefani et al., 2012; Shi et al., 2014; Williamson et al., 2020; Levoux et al., 2021). Mitochondria feature a bidirectional membrane structure with a smooth external membrane and an inner membrane that folds inward to produce a ridge-like structure filled with matrix. Live cell imaging techniques have helped uncover the dynamic alterations of mitochondria, including changes in their form, length, and distribution in cells *via* continual fusion, fission, selective destruction and transit (Lefebvre et al., 2021). These activities are collectively referred to as “mitochondrial dynamics”, which can regulate the quantity, size, and location of mitochondria in the cytoplasm. Mitochondrial dynamics is directly associated with cell homeostasis (Zemirli et al., 2018). For example, stimulation of cells by a variety of internal and external environmental factors (inflammation, pathogen infection, oxidative stress), can cause damage to the internal components of the mitochondria (Stavru et al., 2011; Wu et al., 2011; Zhao et al., 2021). Mitochondrial fusion enables the exchange of material between different mitochondria, while the fission process enables separation of damaged mitochondrial components, thereby achieving a “mitochondrial quality control” effect (Twig et al., 2008). Similarly, highly damaged mitochondria can be destroyed by the ubiquitin protease hydrolysis pathway or encapsulated in autophagic vesicles for lysosomal destruction (Lieber et al., 2019). Mitochondrial fusion occurs when the cellular energy demand exceeds the cellular production of ATP; this phenomenon occurs both physiologically, as with the enhanced skeletal muscle cell function following prolonged exercise (Ruegsegger and Booth, 2018; Arribat et al., 2019), and pathologically, as with the metabolic reprogramming of liver tumor cells (Li et al., 2020).

*Chlamydia* is a common pathogen that causes disease in humans or animals. The organism lacks the capacity to create growth and development products (Stephens et al., 1998); therefore, utilization of host high-energy metabolites (such as ATP and GTP) are essential for its growth and reproduction. Hence, *Chlamydia* is sometimes referred to as “energetic parasite” (Chowdhury and Rudel, 2017). It undergoes a unique biphasic developmental cycle from elementary body (EB) to reticulate body (RB); EB is resistant to the harsh external environment and is infectious, while RB has a high metabolic activity (Tan et al., 2012a; Bastidas et al., 2013). The *Chlamydia* cell cycle may be roughly classified into three stages, as shown by *Chlamydia trachomatis*. Phase 1 (6–8 hours after infection) involves EB to RB transition, early gene transcription, early effector modification of the inclusion membrane, and capture or incorporation of host lipid vesicles (Tan et al., 2012b; Moore and Ouellette, 2014). Phase 2 (8-16 hours after infection) involves the expression of mid-stage genes, which results in the translation of effector proteins that regulate nutrition uptake and host cell survival; in addition, the bacteria divide by binary fission and there is substantial expansion of inclusion (Elwell and Engel, 2012; Tan et al., 2012c). Phase 3 (24–72 hours after infection): late gene expression, RB to EB transition, host cell lysis and extrusion of inclusion, release of infectious EBs, and initiation of a new infection cycle (Tan et al., 2012b). Studies have revealed that changes in host cell mitochondrial dynamics are associated with ATP acquisition, mitochondrion-fatty acid interplay during persistent infection (Shima et al., 2021), and inhibition of host cell inflammatory responses during the Chlamydial cell cycle; all of these biological behaviors are key nodes in the chlamydial cell cycle (Dzakah et al., 2021), indicating a close relationship between *Chlamydia* infection and host cell mitochondria. Therefore, in-depth investigation of the link between C*hlamydia* infection and mitochondrial dynamics is a key imperative for the prevention and treatment of C*hlamydia*-related disorders.

In this review article, we first discuss the molecular processes of mitochondrial fusion and fission. Then, we discuss the influence of *Chlamydia* infection on mitochondrial dynamics and the possibility of suppressing *Chlamydia* development by modifying mitochondrial dynamics. In the future, mitochondrial dynamics might become a novel target for anti-chlamydial infection therapy.

## Mitochondrial Fusion and Fission Dynamics

### Mitochondrial Fusion

There are two types of mitochondrial fusion: inner mitochondrial membrane fusion and outer mitochondrial membrane fusion. Both types of mitochondrial fusion occur concurrently and in unison. Mitofusin-1 (MFN1), mitofusin-2 (MFN2), and ocular atrophy protein 1 (OPA1) all play critical roles in mitochondrial fusion (Chen et al., 2003; Wai et al., 2015). The fusion process comprises of 3 stages: To begin, the outer mitochondrial membrane fusion proteins MFN1 and MFN2 interact with each other resulting in a decrease in the distance between two mitochondria (Qi et al., 2016). Then, MFN1 and MFN2 form a dimer complex to ensure that dynamin-related GTPase mediates the fusion (Cao et al., 2017). Finally, the OPA1 protein is involved in the fusion of the inner mitochondrial membrane (Meeusen et al., 2006).

**Figure 1:**
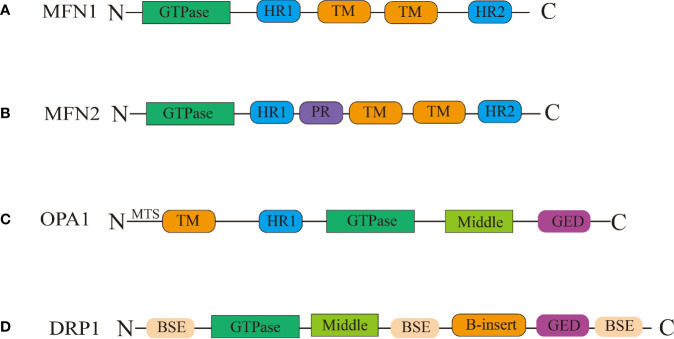
Schematic representation of the structural elements of the fission and fusion proteins. **(A)** Between the heptad repeat 1(HR1) and heptad repeat 2 (HR2) domains, MFN1/MFN2 have two transmembrane (TM) domains Between the HR1 and HR2 domains. **(B)** MFN2 contain proline rich (PR) domains in between HR1 and TM domains. **(C)** OPA1 have been shown to have five domains: the TM domain, the HR1 domain, the GTPase domain, the middle domain, and the GTPase effector domain (GED) domain, the mitochondrial targeting sequence(MTS) located in the N-terminal. **(D)** The DRP1 protein contains the following domains: the bundle signalling elements (BSE) domain, the GTPase domain, the middle domain, the variable domain (or B-insert), the GED domain.

**Figure 2:**
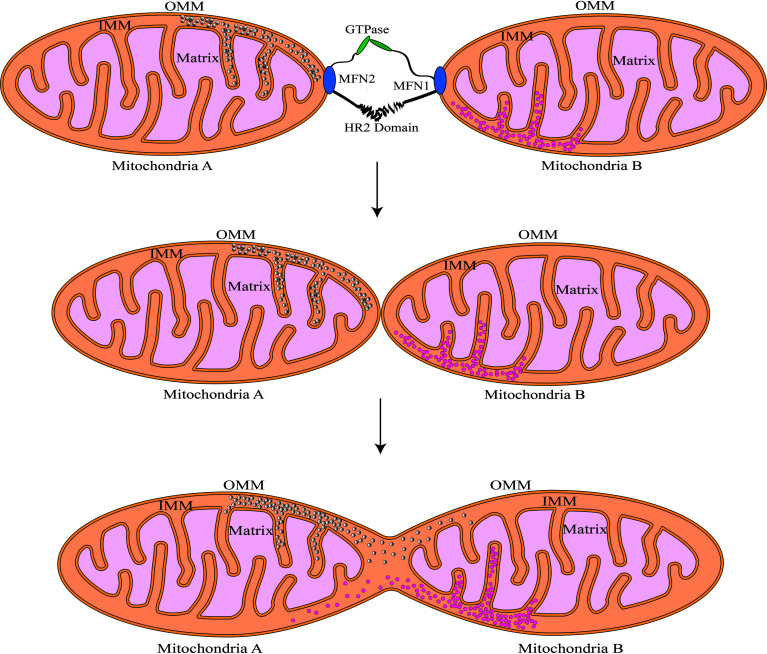
Schematic illustration of fusion of the mitochondrial outer membrane. MFN1/2 forms dimers by linking the hydrophobic heptapeptide repeat structural domains and increases outer mitochondrial membrane cohesion through GTPase hydrolysis. After the outer membrane fusion, Mitochondria A and B can share the gap’s contents.

MFN1 and MFN2 are critical regulators of mitochondrial outer membrane fusion. MFN1/MFN2 are GTPase-mediated motility proteins that are abundantly found in mammals (Song et al., 2009). Both these proteins include four major domains: the conserved GTPase catalytic binding domain at the N-terminus, the structural domains of the heptad repeat 1 (HR1) and heptad repeat 2 (HR2), and the C-terminal transmembrane structure domain (**Figure 1**) (Casellas-Diaz et al., 2021). MFNl/2 are capable of forming dimers by connecting hydrophobic heptapeptide repeat structural domains and facilitate outer mitochondrial membrane cohesion through GTPase hydrolysis, in which the fusion mitochondria share adenylate kinases, metabolites, and proteins (Meeusen et al., 2006; Song et al., 2009; Qi et al., 2016; Cao et al., 2017; Casellas-Diaz et al., 2021). A schematic illustration of this procedure is shown in **Figure 2**.

**Figure 3:**
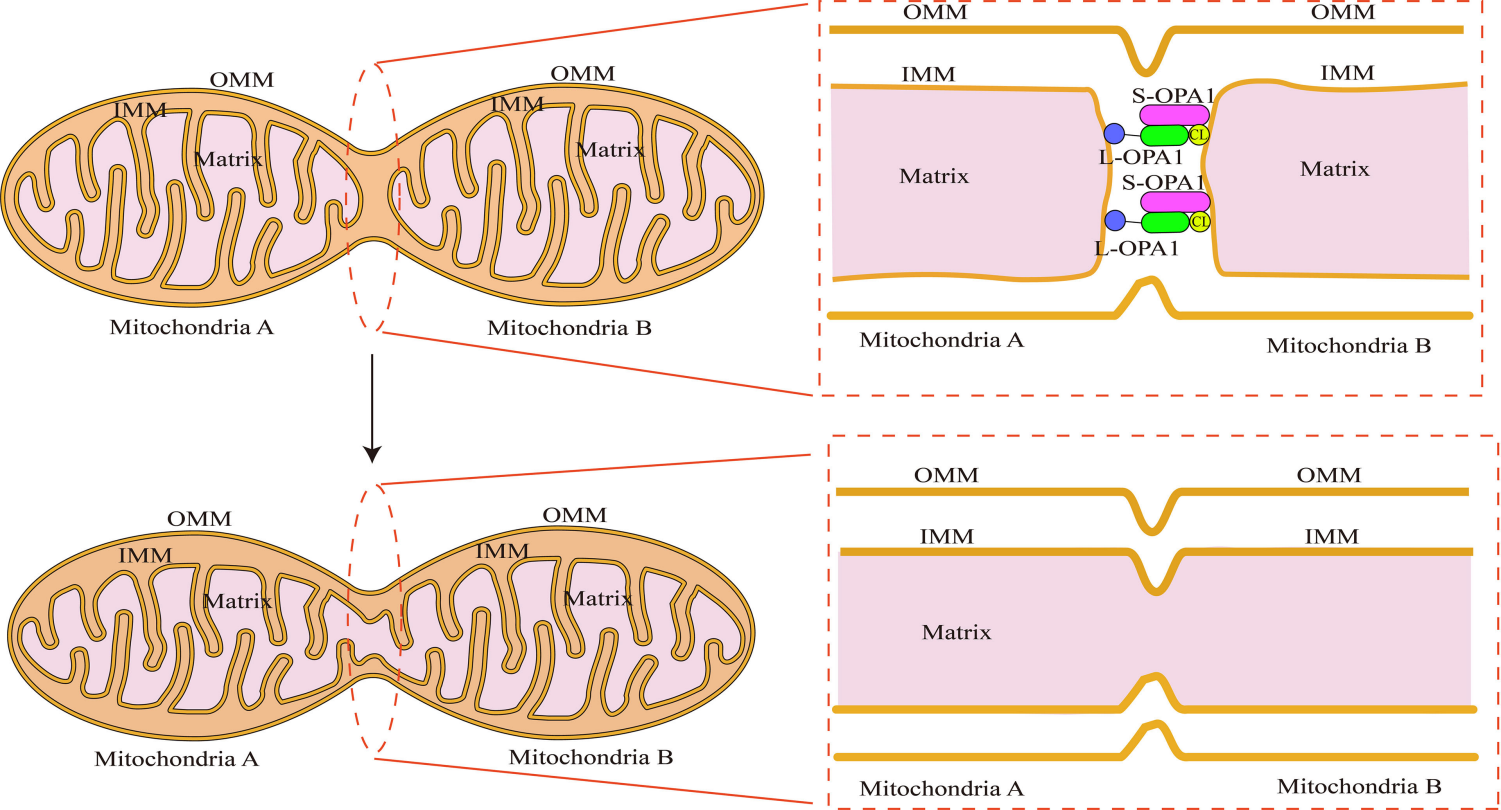
Schematic illustration of fusion of the mitochondrial inner membrane: OPA1’s N-terminal binds to the inner mitochondrial membrane. The membrane gap exposes the GTPase functional structural domain. In a millisecond, OPA1 completes head-tail assembly, creating membrane bending and unstable tips on the two opposing endosomes with the help of cardiolipin (CL). Preparing for endosomal fusion. When two unstable tips meet, the plasma membranes fuse, generating a small hole that connects mitochondria A and B. The contents of the two mitochondrial matrices can be exchanged.

In comparison to outer membrane fusion, mitochondrial inner membrane fusion is a more complex process. OPA1 is a protein involved in the remodeling of the mitochondrial inner membrane that was first discovered in the autosomes of individuals with hereditary ocular atrophy disease (Eiberg et al., 1994). OPA1 is required for the fusion of the inner mitochondrial membrane and the formation of the mitochondrial cristae (Frezza et al., 2006). Not only does OPA1 deficiency result in mitochondrial fragmentation, but it also inhibits the development of mitochondrial inner membrane cristae, which appear as vesicular structures in endosomes under electron microscope (Meeusen et al., 2006). The N-terminal structural domain of OPA1 acts as an anchor, securing it to the inner mitochondrial membrane, whereas the functional structure domain of GTPase is exposed to the membrane gap (Yu et al., 2020). The core machinery proteins are shown in **Figure 1**. On the inner mitochondrial membrane, OPA1 is expressed in two forms: long-form OPA1 (L-OPA1) and short-form OPA1 (S-OPA1). S-OPA1 is generated by hydrolysis of L-OPA1 by OMA1 zinc metallopeptidase (OMA1) and ATP-dependent metal zinc protease (Anand et al., 2014). Neither L-OPA1 nor S-OPA1 alone can stimulate mitochondrial inner membrane fusion, and this process can only occur in the presence of both of them (Song et al., 2007). Although the relationship between OPA1 subtypes and their specific mechanism for mitochondrial inner membrane fusion has not been fully elucidated, it is clear that the ratio of L-OPA1 to S-OPA1 in mitochondrial inner membrane affects the mitochondrial morphology (Wang et al., 2021), implying that S-OPA1 may indeed regulate mitochondrial fusion *via* some mechanism. Hu et al. used the OPA1 protein to create an endosomal fusion model (Gao and Hu, 2021). Due to the fact that OPA1 conducts head-to-tail assembly for a brief amount of time, loading results in membrane bending and the formation of unstable tips on the two opposing inner membranes (Yan et al., 2020). When the head-to-tail assembly structure is broken, this membrane bending phenomenon ceases. When opposing unstable tips come together, lipid mixing results in the formation of fusion holes, and with the progressive expansion of the pore, the mitochondrial inner membrane is completely fused (**Figure 3**).

### Mitochondrial Fission

Mitochondrial fission refers to the division of one mitochondria into two daughter mitochondria. This process is involved in a variety of biological activities, such as organelle inheritance and distribution, mitochondrial distribution, mitochondrial autophagy, and cytochrome C release during apoptosis (Westermann, 2010). Mitochondrial fission in animals is mediated by several proteins such as dynamin-related protein 1(DRP1), Dynamin2(DNM2), mitochondrial elongation factor 2(MIEF2 or MID49), mitochondrial elongation factor 1(MIEF1 or MID51), fission, mitochondrial 1(FIS1), and mitochondrial fission factor(MFF) (Friedman et al., 2011; Schmitt et al., 2018). The DRP1 protein plays a particularly important role in this process (Schmitt et al., 2018). DRP1 is a GTP-dependent kinesin with 5 distinct structural domains (Fröhlich et al., 2013): the bundle signalling elements (BSE) domain, the GTPase domain, the middle domain, the variable domain (or B-insert), the GED domain. DRP1 has bundled signaling components and stalk sections, similar to other kinesins. However, it does not contain the pleckstrin homologous structural domain, the C-terminal proline, arginine-rich structural domain, or the C-terminal of the arginine-rich structural domain (**Figure 1**).

**Figure 4:**
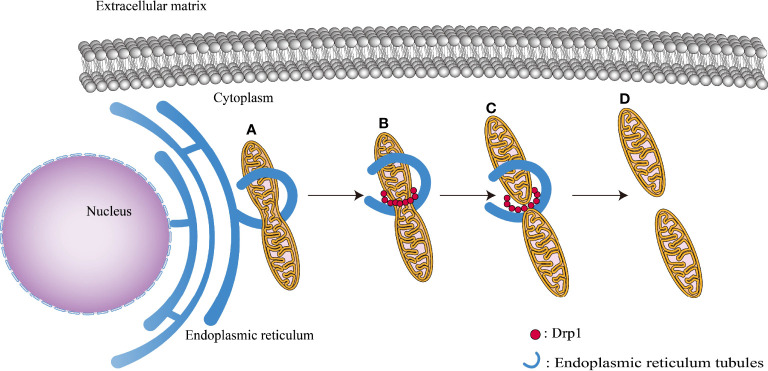
Various phases of mitochondrial fission: **(A)** ER tubules bind to the outer mitochondrial membrane surface, reducing mitochondrial diameter to 150 nm. **(B)** DRP1 recruits to the outer mitochondrial membrane, causing its thinning. **(C)** GTP hydrolysis causes the mitochondrial membrane to contract, preparing the way for the next step of mitochondrial fission. **(D)** Mitochondria completely split.

Mitochondrial fission occurs in a series of steps. The process begins with adherence of the endoplasmic reticulum (ER) tubules to the outer membrane of the mitochondria, shrinking their width from 300–500 nm to 150 nm, providing a spatial foundation for DRP1 oligomerization (Friedman et al., 2011). Subsequently, inverted-formin 2 coupled with the ER tubules interacts with Spire1C protein, initiating the recruitment of DRP1 protein and actin assembly (Korobova et al., 2013; Manor et al., 2015). Then, following recruitment to the outer mitochondrial membrane, DRP1 creates a ring around the mitochondria, which augments the ring contraction force exerted to the mitochondria by ER tubules (Smirnova et al., 2001). Finally, DRP1 hydrolyzes GTP and breaches the outer membrane of the mitochondria (**Figure 4**). It is important to highlight that the inner mitochondrial membrane fission occurs prior to the formation of DRP1 aggregates in the outer membrane; this process is mediated mostly by ER-derived calcium ions (Chakrabarti et al., 2018). Calcium ions enter the mitochondria through the ER and mitochondrial attachment sites, triggering the cleavage of the inner mitochondrial membrane.

Studies have shown that cells can regulate mitochondrial fission by phosphorylating or ubiquitinating DRP1 (Qi et al., 2019; Ma et al., 2020). For example, during mitosis, phosphorylation of the serine residue 585 (S585) of DRP1 results in its oligomerization and attachment to mitochondria, therefore promoting their fission (Taguchi et al., 2007). Additionally, mitogen-activated protein kinase 1 (MAPK1) is required for DRP1 phosphorylation (Song et al., 2021). MAPK1 interacts with DRP1 and phosphorylates serine 616 of DRP1, therefore promoting mitochondrial fission (Kashatus et al., 2015). On the other hand, the biological impact of phosphorylation of the serine residue 637 of DRP1 is opposite to the preceding. Protein kinase A (PKA) has been shown to interact with DRP1 during nutritional deprivation or cell death, retaining it in the cytoplasm and preventing cell lysis and mitochondrial autophagy and degradation (Cribbs and Strack, 2007; Gomes et al., 2011). More modified forms of DRP1 also include SUMOylation, S-nitrosylation, O-GlcNAcylation, and ubiquitination (Jin et al., 2021).

## Effects of *Chlamydia* Infection on Mitochondrial Dynamics

### *Chlamydia* Evades Host Cell Apoptosis by Inhibiting Mitochondrial Fission

Apoptosis is a key mechanism by which cells resist pathogenic infection (Savill and Fadok, 2000). Infected cells that initiate apoptosis on time can reduce pathogen proliferation. Till date, three apoptotic mechanisms are well characterized: receptor-mediated apoptosis (Trauth et al., 1989), mitochondria-mediated apoptosis (Li et al., 1997), and ER stress-mediated apoptosis (Nakagawa et al., 2000). Studies have shown that mitochondrial dynamics-related proteins such as BCL2 associated X(Bax), DRP1, and telomere protein B1 translocate and accumulate from the cytoplasm to the mitochondria early in the initiation of apoptosis, therefore facilitating mitochondrial fission (Martinou and Youle, 2006; Suen et al., 2008). The outer mitochondrial membrane permeability is altered after mitochondrial fission, resulting in release of cytochrome C into the cytoplasm, which permanently activates the caspase signaling pathway and causes apoptosis (Frank et al., 2001). The impact of host cell apoptosis on chlamydial growth and development is evident; premature host cell death leads to nutrition loss and blocks its reproduction. *Chlamydia*, on the other hand, seems to have evolved ways to resist apoptosis in the host cell throughout time (Sixt et al., 2017; Weber et al., 2017; Kerr et al., 2017). *Chlamydia trachomatis* inhibits mitochondrial fragmentation by altering the structure of host cell mitochondria. In a study, after stimulation with H_2_O_2_, mitochondria infected with *Chlamydia trachomatis* did not fragment as much as those in the uninfected group, and the mitochondria grew in length and developed cross-linkages to form a complex network (Chowdhury et al., 2017). In addition, mitoCRWLR, a unique macro script, was utilized to identify changes in mitochondrial movement rate in the infected cells, which is a reliable test for mitochondrial fusion/fission ratio (Okamoto and Shaw, 2005). The findings revealed that stimulation with H_2_O_2_ caused severe fragmentation of the mitochondria of uninfected cells and increased the frequency of random mitochondrial movements; however, cells infected with *Chlamydia* were able to withstand such alterations (Chowdhury et al., 2017). By blocking mitochondrial fission, *Chlamydia* infection avoids the early commencement of the apoptotic pathway, eventually creating a favorable survival environment for itself. This shows that interfering with *Chlamydia*’s molecular processes that impact the mitochondrial dynamics is a potential target for the treatment of *Chlamydia* infection.

### *Chlamydia* Promotes Mitochondrial Fusion to Meet Energy Needs

The intermediate stage of chlamydial growth is characterized by extensive replication of RBs. The energy necessary for RB replication is sourced from the host cells (Omsland et al., 2012), which results in a significant “ATP deficit” in the host cells. *Chlamydia*, on the other hand, seems to have developed techniques to influence the metabolic processes of host cells in order to boost the availability of resources for its own use (Wang et al., 2017). *Chlamydia* can increase the energy output of the host cell by altering mitochondrial dynamics: Kurihara et al. observed that the cellular oxygen consumption of HeLa cells continued to increase following infection with *Chlamydia trachomatis*, and the oxygen consumption surpassed the predicted limit after 6-7 hours (Kurihara et al., 2019). Furthermore, they discovered that the increase in cellular ATP generation rate corresponded with mitochondrial fusion, which was validated by knocking down genes associated with mitochondrial fusion proteins. This seems to imply that *Chlamydia* promotes mitochondrial elongation as a means of obtaining more ATP. Previous research has demonstrated that variations in cell energy needs (ADP/ATP ratio) influence the mitochondrial dynamics, and mitochondrial elongation may also increase cell productivity (Molina et al., 2009).However, whether the modification of mitochondrial dynamics is just a phenomenon or a consequence of chlamydial infection is not clear. Further studies are required to determine whether the outcome is due to the influence of the change in the cellular ADP/ATP ratio or to the active action of *Chlamydia*.

## Mechanisms of Mitochondrial Dynamics Induced by Chlamydial Infection

Mitochondria are in a perpetual state of a highly dynamic process of fusion and fission that is intimately connected with the cell cycle, immunity, apoptosis, and mitochondrial quality control. Numerous variables may influence mitochondrial fusion and fission, including reactive oxygen species (ROS), intracellular metabolic activity, and changes in mitochondrial proteins (Bota and Davies, 2002; Molina et al., 2009; Watanabe et al., 2014). *Chlamydia* infection has been shown to alter the mitochondrial dynamics of host cells in general (Chowdhury et al., 2017). As an exclusively intracellular parasitic bacteria, this change in mitochondrial dynamics is likely critical to the developmental cycle of the bacterium. In-depth characterization of the underlying mechanisms of the altered mitochondrial dynamics associated with chlamydial infection is critical for prevention and therapy.

Initially, ROS were believed to be metabolic byproducts of mitochondrial oxidative phosphorylation (Yang et al., 2016). Increasing evidence indicates that ROS may operate as signaling molecules in a variety of organismal life processes, including inflammation, death, and cell cycle (Moloney and Cotter, 2018). Indeed, ROS-induced changes in mitochondrial dynamics are closely connected; several studies have shown that ROS in mitochondria may affect the state of mitochondrial fusion and fission through their influence on mitochondrial dynamin (Chuang et al., 2020). *Chlamydia* enters host cells and releases virulence factors through type III secretion system(T3SS) effectors, resulting in potassium ion efflux across the cell membrane, activation of the nicotinamide adenine dinucleotide phosphate (NADPH) oxidase, and formation of ROS (Abdul-Sater et al., 2010). It is worth noting that mitochondria, not NADPH oxidase, are the principal source of intracellular ROS. NADPH oxidase-produced ROS acts more like a signaling spark that lays the scene for subsequent mitochondrial ROS generation. Initially, ROS produced in the cytoplasm by NADPH oxidase activates the NOD-like receptor X1 (NLX1) protein, a member of the NOD family that is normally localized to the outer mitochondrial membrane (Tattoli et al., 2008; Nagai-Singer et al., 2019). This induces the translocation of NLRX1 to the inner membrane and its binding to mitochondrial complex III, thereby inhibiting electron transfer and ultimately increasing mitochondrial ROS with mitochondrial depolarization (Abdul-Sater et al., 2010). Available evidence suggests that mitochondria and ER are significant sources of intracytoplasmic calcium ions (Ishii et al., 2006), and mitochondrial depolarization increases intracytoplasmic calcium ions, activating the cytoplasmic phosphatase calcium-regulated phosphatase; this induces phosphorylation of the serine residue 585 of DRP1, resulting in its activation (Bustillo-Zabalbeitia et al., 2014; Macdonald et al., 2014). Activated DRP1 translocates to the outer mitochondrial membrane and promotes mitochondrial fission (Taguchi et al., 2007). Additionally, ROS generated by chlamydial infection may influence mitochondrial dynamics by deactivating ROS modulator 1 (ROMO1), a protein that modulates the function of the OPA1 protein (Norton et al., 2014). Thus, during the early stages of chlamydial infection (0-6 h), there is increase in mitochondrial ROS (mtROS) (Kurihara et al., 2019). However, chronic increase in mtROS has adverse consequences such as apoptosis and cellular energy starvation, which are clearly unfavorable for chlamydial growth (Zhang et al., 2019). Thus, mtROS synthesis is restricted during the mid-stage of the chlamydial growth cycle (Chowdhury et al., 2017), when *Chlamydia* has a high need for energy. However, the specific mechanism by which mtROS is inhibited during the intermediate stage of chlamydial infection is not clear. Given the intimate relationship between NLRX1 and mitochondrial ROS generation, we hypothesize that some phenomena occurs during the middle stage of chlamydial infection that restricts the translocation of NLXR protein to the mitochondrial inner membrane, reducing mtROS production. This is an important subject for future research.

Interestingly, when *Chlamydia pneumoniae* infects host cells, the mitochondrial destiny seems to reverse—mitochondrial dysfunction, as seen by decreased ATP generation and increased ROS production (Kading et al., 2017). Additionally, the researchers discovered that mitochondrial dysfunction promotes *Chlamydia pneumoniae* proliferation and development. It seems to contradict prior research findings that *Chlamydia trachomatis* infection boosts mitochondrial fusion and ATP generation in host cells. Thus far, reasonable explanations can be offered: Mitochondrial dysfunction promotes increased ROS release, which can act as upstream signaling molecules activating caspase-1 and hypoxia-inducible factor-1 (HIF-1) (Prusty et al., 2012), thereby promoting the development of *Chlamydia trachomatis* and possibly also *Chlamydia pneumoniae* growth. Additionally, Different metabolic characteristics might occur due to the different tissue tropism and differences in their genome, which requires an individual adaption of the two species (Kading et al., 2017). These speculations still need follow-up *in vivo* experiments to confirm.

*Chlamydia* and mitochondria have a close interaction, which is maintained by post-translational modification of the DRP1 protein. Recent research has shown that *Chlamydia trachomatis* induces an increase in intracellular cyclic adenosine monophosphatec(AMP) during the early stages of infection, followed by the phosphorylation of cleavage-inactive serine residue 637 (S637) of DRP1 (Kurihara et al., 2019). This eventually results in mitochondrial fusion. Chlamydial infection induces a significant downregulation of the tumor suppressor P53 *via* murine double minute2(Mdm2) regulation of the phosphoinositide 3-kinase Akt (Siegl et al., 2014). Because P53 is a critical regulator of DRP1 dephosphorylation, its downregulation directly affects the activation of DRP1, which eventually manifests as mitochondrial fusion. Additionally, in a study by Rudel et al, infection of human umbilical vein endothelial cells with *Chlamydia trachomatis* was found to induce downregulation of P53 mRNA, resulting in decreased protein expression of P53 in the cytoplasm and decreased phosphorylation of DRP1, inhibiting mitochondrial fission; this change was caused by overexpression of miRNA-30c-5p in the host cells (Chowdhury et al., 2017).

Tail-anchored MFF is an upstream regulator of DRP1 that is responsible for the preferential recruitment of DRP1 protein to the outer mitochondrial membrane (Liu and Chan, 2015), MFF deregulation can result in a decrease in the quantity of DRP1 in the outer mitochondrial membrane, resulting in mitochondrial elongation. On the other hand, overexpression of MFF may result in considerable mitochondrial fragmentation, which is a necessary condition for mitochondrial fission (Loson et al., 2013). Myeloid cell leukemia sequence 1(Mcl-1) acts as an anti-apoptotic protein (Huang et al., 2019), inhibiting the oligomerization of DRP1 protein mediated by MFF protein and preventing mitochondrial fragmentation, hence acting as an anti-apoptotic protein (Liu et al., 2020). *Chlamydia* increases the degree of Mcl-1 deubiquitination and the cytoplasmic concentration of Mcl-1 protein, consequently limiting mitochondrial fission (Fischer et al., 2017). In host cells infected with *Chlamydia*, the relevant genes initiate transcription and translation of the ChlaDub1(Cdu1) protein expressed on the inclusion membrane; the subsequent accumulation of the Mcl-1 protein in the cytoplasm results in the formation of Cdu1-Mcl-1 complex which directly protects Mcl-1 from K48-ubiquitinated degradation (Pruneda et al., 2016).

**Figure 5:**
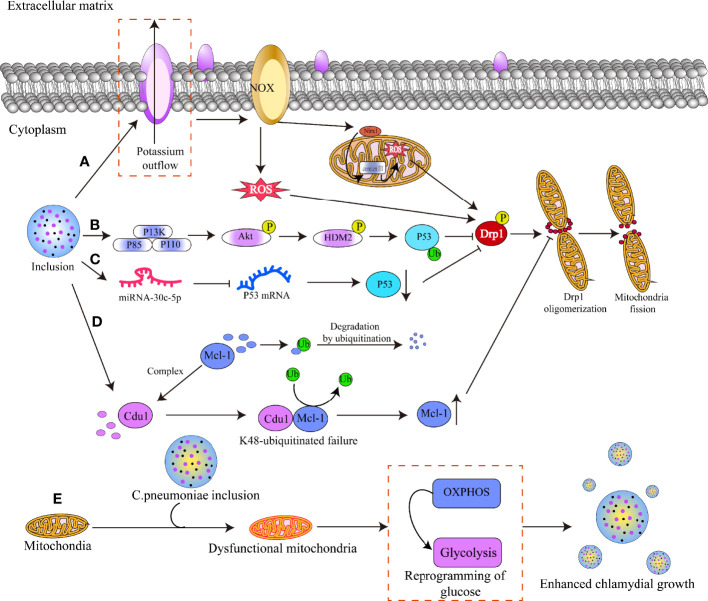
Mechanisms by which chlamydial infection alters mitochondrial dynamics. **(A)** *Chlamydia* infects host cells and releases virulence factors through T3SS effectors, resulting in potassium efflux from the cell membrane and activation of the NADPH oxidase. This induces the production of reactive oxygen species (ROS) in the cytoplasm, which acts as a secondary signal to activate the NLRX1 protein in the outer mitochondrial membrane. The activated NLRX1 protein translocates and binds to mitochondrial complex 3, resulting in massive mitochondrial ROS production and phosphorylation of the serine residue 5 8 5 of DRP1, activating DRP1. **(B)** *Chlamydia* triggers the PI3K-ATK-HMD2 signaling pathway, which results in the ubiquitination of the P53 protein and subsequently suppresses the activation of the DRP1 protein. **(C)** *Chlamydia* increases host cell miRNA-30c-5p production, which binds to the mRNA of the P53 protein and blocks its translation, hence inhibiting DRP1 protein activation. **(D)** *Chlamydia* produces the cdu1 protein, which binds to Mcl-1 and shields it from ubiquitinated degradation, hence raising Mcl-1 protein levels and preventing DRP1 oligomerization. **(E)** C. pneumoniae infects host cells, resulting in mitochondria dysfunction and switch of OXPHOS to glycolysis, which enhances the bacteria growth.

Thus, *Chlamydia* suppresses DRP1 protein function *via* a variety of mechanisms, inhibiting mitochondrial fission (**Figure 5**). As we shall detail below, impeding *Chlamydia* reproduction in host cells by interfering with mitochondrial dynamics seems to be a novel and extremely promising treatment strategy.

## Mitochondrial Dynamics: A Potential Therapeutic Target

Development of drugs that regulate mitochondrial fusion, fission, and autophagy is a contemporary research hotspot, indicating that mitochondrial dynamics is a potential therapeutic target for many diseases (Jheng et al., 2015; Anis et al., 2020; Axelrod et al., 2021). For example, mitochondrial fission inhibitor-1 (Mdivi-1), a GTP synthase inhibitor, has been studied for its ability to limit mitochondrial function by reducing mitochondrial mass, inducing apoptosis, reducing vascular cell bioenergetics, and inhibiting cell proliferation (Kim et al., 2013). Because of its capacity to prevent cell growth, Mdivi-1 has long been considered a potential cancer therapy. Similarly, after infecting host cells, *Chlamydia* needs to prevent apoptosis in order to maintain a favorable survival environment, at least in the early and intermediate phases of infection. Tumors are similar in their method of interfering with the natural apoptotic process of cells. Thus, mdivi-1 may be useful in the treatment of chlamydial infection.

On the other hand, chlamydial infection induces activation of the phosphatidylinositol-3-kinase(PI3K)-autologous tumor killing(ATK)-Mdm2 signaling pathway in order to maintain low intracellular P53 levels (Zou et al., 2019), which further limits the activation of DRP1 and results in widespread host cell mitochondrial fusion. As a result, PI3K inhibitors may have therapeutic utility. For example, use of a PI3K inhibitor (LY294002) was investigated in an *in vitro* study of chlamydial infection (Siegl et al., 2014), in this study, PI3k inhibition dramatically stabilized P53 levels in infected human umbilical vein endothelial cells. Likewise, Al-Zeer et al. discovered that chlamydial infection can result in phosphorylation of the serine residue 637 of pyruvate dehydrogenase kinase 1(PDK1) in host cells, hence stabilizing the production of WYC protein. Consistent MYC expression can induce hexokinase II(HKII) upregulation (Al-Zeer et al., 2017). This is critical for cell survival, because the mitochondria-HKII association favorably influences mitochondrial energetics and cell survival by preventing pro-apoptotic Bak and Bax oligomerization and binding at the level of the mitochondrion (Robey and Hay, 2006). It is also crucial for inhibiting cytochrome c release and apoptosis, which is controlled by the PI3K cascade (Kennedy et al., 1999). Thus, genetic and pharmacological means to inhibit MYC and 3-Phosphoinositide Dependent Protein Kinase 1(PDPK1) may also block chlamydial replication. TH2-mpeoDM1(HMD2) is a critical component in the ubiquitination and degradation of the P53 protein. HDM2 interacts with P53 in the nucleus, facilitates its export to the cytoplasm, and catalyzes the production of ubiquitin chains on P53 (Bhatt et al., 2012). In other words, suppressing the interaction between HMD2 and P53 may result in stabilization of P53 protein level in cells. Nutlin-3, a cis-imidazoline, selectively binds to HDM2 and competes with P53 protein, limiting the binding of HDM2 to the N-terminal end of P53 (van Leeuwen et al., 2011), but still maintaining intracellular P53 levels, facilitating mitochondrial fission.

Additionally, phosphorylation is a significant post-translational alteration of the DRP1 protein. Numerous phosphorylation modification sites on the DRP1 protein have been identified so far, including Ser-579, Ser-40, Ser-585, Ser-44, Ser-592, Ser-656, Ser-616, Ser-637, and Ser-693 (Qi et al., 2019). Among these, phosphorylation of Ser-616 on DRP1 may activate the protein and cause mitochondrial fission. Rho-associated protein kinase (ROCK), protein kinase C delta (PKC), cyclin-dependent kinase 1 (CDK1), extracellular signal-regulated kinase 1/2 (ERK1/2), and calmodulin-dependent protein kinase II (CaMKII) were discovered to catalyze this site (Bo et al., 2018; Adaniya et al., 2019). Unlike the Ser-616 location, phosphorylation of the serine residue 637 of DRP1 results in a decrease in GTPase hydrolysis activity (Chang and Blackstone, 2007; Cribbs and Strack, 2007). That is, phosphorylation of this region results in a decrease in DRP1 activity. In theory, regulating the expression of the above enzymes can indirectly affect the phosphorylation of the serine Ser-616 and Ser637 of DRP1, activate DRP1 protein, promote mitochondrial fission events, and ultimately disrupt the environment in which *Chlamydia* survives.

Finally, *Chlamydia*’s unique “Cdu1-Mcl-1” interaction mechanism, which results in Mcl-1 deubiquitination, is another potential therapeutic target. Cdu1-Mcl-1 binding was shown to decrease Mcl-1 ubiquitination and hinder DRP1 protein oligomerization, preventing mitochondrial fission and enhancing mitochondrial fusion (Fischer et al., 2017). Thereby, it seems to be an extremely promising strategy to create ligands that exclusively bind to Cdu1 as a competitive inhibitor of Mcl-1 in order to increase the ubiquitination level of Mcl-1 and thus impact mitochondrial dynamics. Similar investigations are currently ongoing: Caroline Kisker’s team discovered that the particular inhibitors cyanopyrimidine 3 and cyanopyrimidine 5 may form covalent connections with Cdu1 and disrupt the protein’s deubiquitination function (Ramirez et al., 2018). This indicates that the chemical may be capable of combating chlamydial infection.

However, it is critical to consider drug targeting when using mitochondrial dynamics as a therapeutic target for chlamydial infection. As a rule of thumb, it is preferable to have more frequent mitochondrial fission events in certain infected cells rather than all cells of the organism; otherwise, it may result in adverse effects such as decreased collective energy metabolism and increased apoptosis. The question of how to design pro-mitochondrial fission drugs that act only in chlamydial infected cells deserves to be researched in greater depth.

## Concluding Remarks and Future Perspectives

Mitochondria are ephemeral organelles with a high degree of dynamic activity. The homeostasis between fusion and fission is critical for cell activities. This has an effect on not just ATP synthesis, but also on the classic apoptotic pathway. Chlamydial infection affects the mitochondrial dynamics, and altered mitochondrial dynamics can have an effect on *Chlamydia* survival. *Chlamydia* inhibits mitochondrial fission, preventing the host cell from commencing the apoptotic pathway and enabling the cell to continue its cycle. Additionally, *Chlamydia* controls the efficiency of energy generation by the host cells by stimulating mitochondrial fusion to satisfy self-replication and reproduction requirements. *Chlamydia* suppresses mitochondrial fission and promotes mitochondrial fusion in the host cell *via* lowering ROS generation, inhibiting P53 protein transcription, increasing P53 protein ubiquitination levels, and inhibiting DRP1 protein activity. These mechanisms are potential therapeutic targets. This review explored the effect of chlamydial infection on mitochondrial dynamics and the underlying mechanisms. We also reviewed the available evidence pertaining to other drugs that impede mitochondrial fusion on this basis, including mdivi-1, LY294002, Nutlin-3, cyanopyrimidine 3, and cyanopyrimidine 5. Although medication targeting remains an open question, with breakthroughs in research on drug release and delivery systems, pharmacological targeting of the mitochondrial dynamics of infected cells seems to be a viable therapeutic strategy for chlamydial infection.

## Author Contributions

Manuscript conceptualization, ZL and YY. Writing original manuscript draft, YY. Literature search and articles acquisition, YW and LZ. Figures drawing, WL. All authors contributed to the article and approved the submitted version.

## Funding

This work was supported by the National Natural Science Foundation of China (No. 32070189 and 81772210), the Key Program of Hunan Provincial Department of Education (No. 20A421), Hunan Provincial Natural Science Foundation of China (No.2021JJ30594), Clinical Research Project of University of South of China (No. USCKF201902K01).

## Conflict of Interest

The authors declare that the research was conducted in the absence of any commercial or financial relationships that could be construed as a potential conflict of interest.

## Publisher’s Note

All claims expressed in this article are solely those of the authors and do not necessarily represent those of their affiliated organizations, or those of the publisher, the editors and the reviewers. Any product that may be evaluated in this article, or claim that may be made by its manufacturer, is not guaranteed or endorsed by the publisher.
